# UnionPromptNER serves as a union prompting method to bridge few-shot named entity recognition

**DOI:** 10.1038/s41598-025-30822-8

**Published:** 2025-12-02

**Authors:** Wei Tian, Shuangshuang Xu, Yongwei Wang, Hao Li, Hao Zhu

**Affiliations:** 1 School of Civil Engineering, Southeast University, Mozhou East Road, Nanjing, Jiangsu 211100 China; 2 Intelligent Construction Technology Department, CCCC Second Harbor Engineering Co., Ltd., Chunxiao Road, Wuhan, Hubei 430200 China

**Keywords:** Few-shot named entity recognition, UnionPromptNER, Prompt strategy, Label semantic, Computational science, Computer science, Information technology, Statistics

## Abstract

The task of few-shot named entity recognition (NER) is to identify named entities by using limited annotated samples. Meta-learning, as a specific paradigm in the field of machine learning, has shown good results in acquiring the ability to “learn how to learn” and in quickly learning new tasks. However, some methods in the field of meta-learning identify named entities by calculating the word-level similarity between the query set and support set, without fully considering the label semantic information. To address this issue, we propose a method called UnionPromptNER for few-shot named entity recognition in the bridging domain. This method utilizes a joint prompt strategy to acquire label semantics, and then introduces a framework for computing the semantic representation of joint prompts. Through experiments on three different types of datasets, our proposed method achieved the best results in 19 out of 20 different settings compared with a series of previously optimal methods based on the micro F1 metric.

## Introduction

**Fig. 1:**
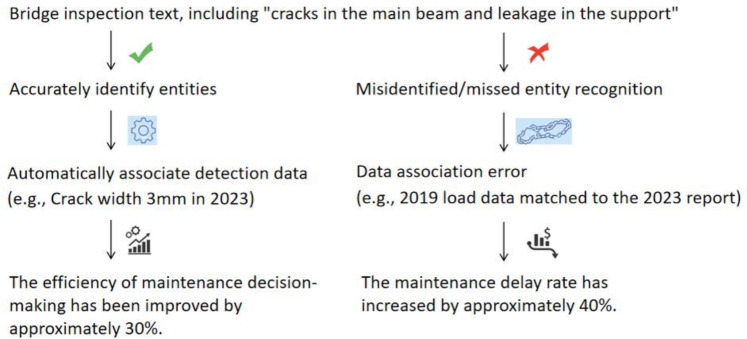
Schematic diagram of the function of the NER module in the bridge domain.

As an essential component of the transportation infrastructure, bridges play a pivotal role in fostering economic development and enhancing the quality of human life. Numerous bridges have been constructed globally. For instance, at the end of 2022, China had 1,032,200 road bridges with a total length of 85.7649 million linear meters. Among them, there are 8,816 super-sized bridges with a total length of 16.2144 million linear meters, and 15.96 thousand large bridges with a total length of 44.3193 million linear meters.During the construction of road bridges, the industry has accumulated a large amount of textual data, including construction plans, regulatory references, and various types of contracts.These texts contain a wealth of entity and relationship information, which must be explored. Research on key information extraction methods based on named entity recognition in bridge texts can provide core data support for generating specialized plans, intelligent decision-making during the operation of road bridges, and knowledge sharing in the field. This is also an important trend in the digitalization and intelligent development of the civil engineering industry. Figure 1 illustrates the corresponding results when the entities of the NER module in the bridge domain are correctly recognized and when they fail to be correctly recognized.

Named entity recognition (NER), a fundamental task in information extraction, is dedicated to extracting entity information from unstructured text and categorizing it into relevant classes. It has received wide attention and significant development in research fields such as machine learning, deep neural networks, and natural language processing. However, it is important to note that text related to bridges exhibits distinct domain-specific characteristics in terms of specialized terminology, writing style, and structural organization. General domain named entity recognition methods, which typically focus on identifying person names, organization names, and place names, may not yield optimal performance when applied to the bridge domain. The differences between general domain data and bridge domain data in three aspects of professional terminology are shown in Table 1. Additionally, during the process of named entity recognition, the availability of annotated data specifically tailored to the bridge domain is limited, and manual annotation is both time-consuming and expensive. Hence, it is imperative to investigate a named entity recognition solution that is well-suited for the limited sample scenarios encountered in the bridge domain.

**Table 1:** The differences between general domain data and bridge domain data in three aspects of professional terminology.

Dimensions of comparison	General domain data	Bridge domain data
terminological features	It centers on “generalization and popularization” and rarely uses professional terms; even when terms are involved, they are mostly basic words familiar to the public (such as “speed”, “weight”, “safety”).	It centers on “professionalization and precision” and is filled with a large number of industry-specific terms, where the terms refer to highly specific professional concepts (such as structure, material, craftsmanship, etc.).
terminological purposes	Lower the threshold for understanding and ensure that audiences with different knowledge backgrounds can quickly access information.	Accurately convey professional information, avoid ambiguity, and meet the need for unified cognition within the industry.
examples	Describing the load-bearing capacity of an object: “This structure can bear very heavy things.” Describing the safety status: “It’s very safe here, so don’t worry.”	Describing structural load-bearing capacity: “The designed bearing capacity of this beam is 2000kN, which meets the requirements of the JTG D60-2015 specification.” Describing the safety status: “The settlement of the support exceeds 15mm, which has gone beyond the allowable value specified in the standard, posing a risk of structural instability.”

In summary, our contributions are as follows:

1. We introduce a bridge text small-sample named entity recognition approach, UnionPromptNER, which based on joint prompt learning, leveraging the information extraction capabilities of pre-trained models.
2. We utilize a a joint prompt strategy to acquire label semantics. Furthermore, we introduce a framework for computing the semantic representation of joint prompts.
3. Experimental results on multiple few-shot NER datasets demonstrate that UnionPromptNER achieves the best results in 19 out of 20 different settings compared with a series of previously optimal methods based on the micro F1 metric.

The remainder of this paper is organized as follows. Section 2 provides an introduction to the current research status of pre-trained models and small-sample named entity recognition in both domestic and international contexts. Section 3 introduces the problem formulation of a few-shot named entity recognition(NER). The proposed method is described in Sect. 4. The next two sections present the experimental setup and results. Finally, conclusions are drawn including limitations and future directions of this study.

## Related work and background

### Pre-training and fine-tuning

Pre-training refers to the process of training a general language model using large-scale datasets, which are then used as an initializer or feature extractor for downstream tasks. In recent years, several large-scale models trained on massive amounts of data have been proposed, such as BERT^1^, GPT^2^, RoBERTa^3^, and T5^4^, which have played significant roles in advancing the field of natural language processing. Prieto et al.^5^ conducted a study in which ChatGPT was used to generate a construction schedule for a simple construction project. Hassan et al.^6^ used a BERT-based model to identify risky and hazardous construction situations. Moon et al.^7^ used a BERT model to automatically detect contractual risk clauses in construction specifications.

The main advantages of pre-training are a reduction in the need for labeled data, improved model generalization, faster training speed, and a reduced risk of overfitting. Fine-tuning refers to using a pre-trained model as the initial parameter and then conducting a small amount of training on a new task to achieve better performance. Fine-tuning language models for large-scale instruction tasks can significantly improve the performance and generalization ability of models in various settings. It is a common and effective approach to enhance the effectiveness and usability of pre-trained language models^8^. Lester et al.^9^ proposed a method for fine-tuning frozen language models by using soft prompts. This prompt tuning method became more competitive as the scale of the model increased. It almost matches the performance of full-model fine-tuning on large models, and has the advantages of robustness and efficiency. Sun et al.^10^ compared full-parameter and LoRA tuning strategies on a Chinese instruction dataset and found that the choice of the base model, the number of learnable parameters, size of the training dataset, and cost are all key factors that affect the performance of instruction-following models.

Pre-training and fine-tuning have become common paradigms for natural language understanding tasks such as named entity recognition, question answering, and text summarization.

### Prompt-based learning

Although pre-training and fine-tuning paradigms have made significant breakthroughs in various natural language processing domains, they have some limitations. Pre-trained models have a large number of parameters and limited flexibility in their model structures. Although fine-tuning can reduce the differences between task distributions, its effectiveness may be limited if the differences are substantial^11^. Additionally, fine-tuning requires maintaining copies of the model parameters for each downstream task during the inference process, which can be inconvenient.

To address these limitations, many researchers have started exploring the use of large-scale pre-trained language models and have developed a new paradigm in natural language processing called “prompting.” Prompting involves leveraging prompts or cues along with a small amount of labeled data to unlock the potential of large models^12,13^. This approach aims to overcome the parameter inflexibility and the limited effectiveness of fine-tuning when dealing with substantial differences in task distributions. Using prompts, researchers can guide the behavior of the model and achieve better performance on specific tasks.

In general, prompting allows pre-trained language models to incorporate additional information when performing downstream tasks^9,14^. According to Petroni et al.^15^, a language model serves as the knowledge base. The LAMA dataset provides manually created completion templates that enable the exploration of knowledge within pre-trained language models. These templates are query sentences designed manually, allowing the extraction of knowledge from the pre-trained language model and achieving similar results compared to using the model directly. However, manually designing templates as discrete prompts can be time-consuming, costly, and requires extensive expertise from designers^16^. Shin et al. introduced gradient-based search methods to automatically create prompts for various tasks, thereby reducing the effort required to construct prompt text^17^. Additionally, some researchers have designed continuous prompts by adding specific prefixes corresponding to small tasks, replacing the need for manual prompt template design and learning through backpropagation^18^. Numerous studies have demonstrated the significant impact of prompt template quality on the model performance.

In summary, prompting-based learning is indeed a worthwhile direction of research, as it not only unlocks the potential of pre-trained language models but also allows for the exploration of their capabilities.

### Few-shot NER

The goal of few-shot named entity recognition (NER) tasks is to classify the identified entities using only a small amount of annotated data. In recent years, several methods have been proposed to address different few-shot learning tasks^14,19–24^ in the NLP community. Sun et al.^25^ proposed an example-based approach for few-shot NER. This approach is inspired by question-answering to identify entity spans in new and unseen domains. Cui et al.^26^ proposed a template-based method for NER, treating NER as a language model ranking problem in a sequence-to-sequence framework, where original sentences and statement templates filled by candidate named entity spans are regarded as the source and target sequences, respectively. Wang et al.^27^ proposed a self-training method for semi-supervised training using a large amount of unlabeled data. A large-scale human-annotated dataset, Few-NERD, was proposed to introduce more fifine-grained entity types in few-shot NER^28^. Chen et al.^29^ proposed a self-describing mechanism that described all entity types as a unified concept set. Mapping between types and concepts can be modeled and learned to address the issue of knowledge mismatch. Current Few-shot NER studies have mostly focused on prompt-based formulations to exploit the Pre-trained Language Model (PLM) knowledge more effectively. To address the issue of knowledge mismatch, Kim et al. designed different label mapping methods to achieve the accurate matching of entities^30^. Beryozkin et al. merged labels of different patterns into the same classification system for knowledge sharing^31^. Guo et al.^32^ proposed a Boundary-Aware LLMs for Few-Shot Named Entity Recognition(BANER) to address the over/under-detected false spans in the span detection stage and unaligned entity prototypes.Tang et al.^33^ introduced data stratification as a preliminary step to consider all entity types fairly and proposed FsPONER which is to incorporate term frequency alongside sentence embedding, enhancing domain-specific NER performance.

We have conducted an extensive and systematic literature review. We searched through major academic databases such as IEEE Xplore, ScienceDirect, SpringerLink, Web of Science, and Google Scholar. The search keywords included combinations of “bridge”, “civil engineering”, “few-shot learning”, “named entity recognition”, “NER”, “limited data”, and related terms. We also reviewed the references of relevant papers to ensure we did not miss any potential studies.

After this thorough investigation, we can confirm that, to the best of our knowledge, there are no existing studies that have specifically focused on few-shot NER in the bridge domain. Previous research on NER in civil engineering or bridge-related fields has primarily relied on large annotated datasets, and few-shot learning approaches have not been applied to this specific domain.

In Contrast to the above related works, we propose a better architectural framework to utilize cue words to mine the semantic information of entities.

## Methodology

**Fig. 2:**
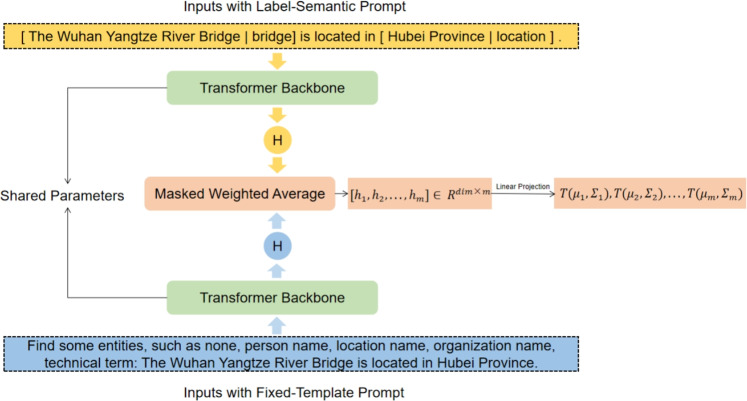
Model structure of our method.

In this section, we formally present the UnionPromptNER scheme. The Architecture of UnionPromptNER is illustrated in Figure 2.

### Problem formulation

**Fig. 3:**
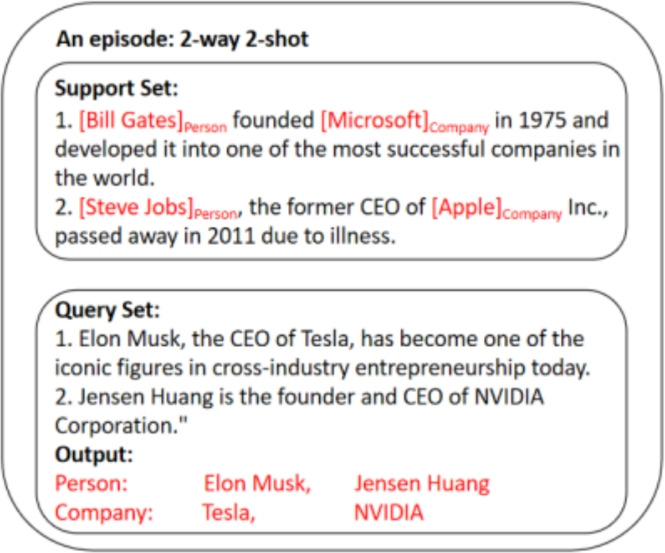
An example of 2-way 2-shot episode.

In this section, we focus on the few-shot NER task. Unlike NER tasks with abundant labeled datasets, few-shot NER tasks require fine-tuning with only a small amount of labeled data. This assumption aligns better with real-world scenarios, because acquiring a large amount of high-quality labeled data often incurs significant costs. Specifically, when training the new dataset *D* with label space *Y*, we assumed that each type had only *K* training examples in the training set. In other words, each type contained *K* data instances with their corresponding true labels. Therefore, the total number of labeled examples is $K_{tot}=K*|Y|$ . Subsequently, the trained model was tested using an unseen test set $(X_{test}, Y_{test}) \sim D_{test}$. In the NER task, a training sample refers to a continuous entity span $e=\{x_{1},...,x_{m}\}$ with a positive class label attached. A typical 2-way 2-shot episode is shown in Figure 3.

### Prompt schemas

Inspired by existing frameworks such as Prompt Learning^26,34^ and Metric Learning, our UnionPromptNER incorporates prompt words to provide label semantics and additional semantic information for the metric-learning model. We propose a simple and effective union prompt word template that allows us to provide different prompt words to the model, which is consistent with metric learning approaches that use token-level similarity as a metric. Starting from this template, we introduced two types of prompt words used in UnionPromptNER: fixed-template prompt words and label-semantic prompt words.

**Prompt A: Fixed-Template Prompts** Typically, the input to an NER system is a natural language sentence. Given a natural language sentence $X=[x_{1},x_{2},...,x_{n}]$ composed of multiple subwords and a set of label categories $L=[none,l_{1},l_{2},...,l_{m-1}]$, where $m=|L|$ and none represents the O-type, we define a fixed prompt word template to restructure the input sentence as follows:

1 $$\begin{aligned} \varvec{X}{p} = \varvec{V}{prompt}(L) \bigotimes X \end{aligned}$$

The template function $\varvec{V}{prompt}(L)$ is used to fill in the template with entity type set, and $X_{p}$ is the result obtained by converting *X* through the template function. For example, if the entity type set is none, person name, location name, organization name, technical term, and the input sentence is “The Wuhan Yangtze River Bridge is located in Hubei Province.” By constructing the fixed prompt word template, the resulting prompt words would be “Find some entities, such as none, person name, location name, organization name, technical term:”, which is then concatenated with the input sentence to obtain the final transformed result: “Find some entities, such as none, person name, location name, organization name, technical term: The Wuhan Yangtze River Bridge is located in Hubei Province.” This transformation, using a fixed prompt word template, provides additional semantic information to the model and guides the model to extract entities based on the prompted words.

**Prompt B: Label-Semantic Prompts** Label semantic prompts are used to add entity labels to the entity words in the input sentence, allowing the model to learn label semantic information. Unlike fixed-prompt word templates, label semantic prompts provide local information to each entity in the model. Specifically, let $V_{B}(x,y)$ be a label semantic-prompt function. For a given input sentence $X=[x_{1},x_{2},...,x_{n}]$ and entity label sequence $Y=[y_{1},y_{2},...,y_{n}]$ , for a certain entity , we first retrieve its corresponding label *x* from the entity label sequence *Y*, and then replace entity *x* in the input sentence with the template $'[x|L]'$ which combines the entity with the label, resulting in the constructed result $x^{'}=V_{B}(x,y)$.

It is worth mentioning that there are other ways to construct prompts, but in practical applications, we found that these two methods performed exceptionally well.

### Model and training

The UnionPromptNER architecture uses a transformer backbone to encode different prompt word results and combines these two types of prompt word results.

During the training phase, we used a small number of samples from the training set $D_{test}$, where each sample was obtained from a few-shot sequence ($\delta _{train}, \varrho _{train}$). We extracted the label set from the support set $\delta _{train}$ and translated each label into a natural language tag by using a lookup table. This results in a set of tags denoted by $\Theta$. For an input sentence $X=[x_{1},x_{2},...,x_{n}]$ and its corresponding label sequence $Y=[y_{1},y_{2},...,y_{n}]$, we obtain the results $r_{A}=V_{A}(x,\Theta )$ and $r_{B}=V_{B}(x,\Theta )$ using fixed template prompt words and label semantic prompt words, respectively.

Next, we fed the prompt word results into a pre-trained language model (PLM) and computed the representations in the intermediate layer as follows:

2 $$\begin{aligned} h_{A} = PLM(r_{A}), h_{B} = PLM(r_{B}) \end{aligned}$$

Next, we combine the two intermediate results using parameter $\rho$ as follows:

3 $$\begin{aligned} h = \rho h_{A} + (1-\rho )h_{B} \end{aligned}$$

Where $\rho \in (0,1)$ is a hyperparameter.

Specififically, we employ projection network $f_{\mu }$ and $f_{\Sigma }$ for producing Gaussian distribution parameters:

4 $$\begin{aligned} \mu _{i} = {f}_{\mu }(h_{i}), \Sigma _{i}=\mathrm ELU( f_{\Sigma }(h_{i})\mathrm ) + (1+\varepsilon ) \end{aligned}$$

Where $\mu _{i} \in \Re ^{l}$, $\Sigma _{i} \in \Re ^{l \times l}$ represents the mean and diagonal covariance of the Gaussian distribution space, respectively. ELU for exponential linear units, and $\varepsilon \approx e^{-14}$ for numerical stability.

In the case of few-shot learning, for word elements $x_{m}$ from the support set and word elements $x_{n}$ from the query set, the Gaussian distributions of the two word elements are as follows:

5 $$\begin{aligned} T_{(\mu _{m}, \Sigma _{m})}, T_{(\mu _{n}, \Sigma _{n})} \end{aligned}$$

The distance between two word elements and their corresponding Gaussian distributions can be calculated using the following formula:

6 $$\begin{aligned} D_{(x_{m}, x_{n})} = \frac{1}{2}(D_{KL}(T_{(\mu _{m}, \Sigma _{m})} || T_{(\mu _{n}, \Sigma _{n})}) + D_{KL}(T_{(\mu _{n}, \Sigma _{n})} || T_{(\mu _{m}, \Sigma _{m})})) \end{aligned}$$

Where $D_{KL}$ means the Kullback-Leibler divergence.

Let $\delta _{train}^{'}$, $\varrho _{train}^{'}$ be collections of all tokens from sentences in $\delta _{train}$, $\varrho _{train}$. For each $q \in \varrho _{train}^{'}$, the associated loss function is computed as:

7 $$\begin{aligned} L(q) = -log \frac{\Sigma _{p \in \chi _q} exp(-D(q,p)) / |\chi _q|}{\Sigma _{p \in \delta _{train}^{'}} exp(-D(q,p))} \end{aligned}$$

Where $\chi _q$ is defined by $\chi _q = \{p \in \delta _{train}^{'}|p,q$ have the same labels}.

To calculate the total loss function for the same batch, which is the sum of the loss functions for all samples, the following process is typically followed:

8 $$\begin{aligned} L = \frac{1}{|\varrho _{train}^{'}|} \Sigma _{q \in \varrho _{train}^{'}} L(q) \end{aligned}$$

### Inference

In the inference phase, for each word element *x* in the query set, the Euclidean distance was calculated based on its representation in the feature space. The word element with the shortest Euclidean distance was selected from all the word elements in the support set, and the corresponding label was returned as the label for word element *x*. This provides an entity label for the word element *x*.

## Experiments

### Experiment datasets

To demonstrate the few-shot learning ability of our method, we conducted experiments on two well-designed N-way K-shot few-shot NER datasets and a bridge-domain construction schema text dataset. **CROSSNER** CrossNER consists of four NER datasets from different domains: CoNLL03^35^(News), OntoNotes^36^(Mixed), WNUT-2017^37^(Social), and GUM^38^(Wiki). **FEW-NERD** Ding et al.^28^ propose a human-annotated low-shot named entity recognition dataset called Few-NERD, which is a large-scale dataset specially designed for few-shot NER with 8 coarsegrained and 66 fifine-grained entity types from Wikipedia.We utilize the IO tagging scheme, where the “O” tag is used for non-entity labeling, and entity tags are assigned corresponding entity type labels. We also converted the annotated abbreviations into plain text, for example, by converting [LOC] to [location]. **Bridge Text Dataset** Bridge Text Dataset were sourced from more than 100 Chinese specifications and construction plan documents in the field of bridges. Following the format of the CoNLL dataset, we prepared two types of low-shot datasets: 1-shot and 5-shot.

### Baselines

For different datasets, we chose different baselines for comparison. For Few-NERD, we compared our approach with recently proposed methods such as DecomposedMetaNER^39^ and ESD^40^. Additionally, the baselines include a contrastive learning method CONTaiNER^41^, a prototypical network, ProtoBERT^42–44^, the nearest neighbor-based network NNShot, and its Viterbi decoding variant StructShot^45^. In addition to DecomposedMetaNER and ProtoBERT, our baselines also include SimBERT, which is a model that predicts labels according to the cosine similarity of word embedding in non-fine-tuned BERT. A classifification head based method, TransferBERT[1], is based on a pre-trained BERT, a few-shot sequence labeling model Matching Network^46^, and a few-shot CRF model for slot tagging of task-oriented dialogue L-TapNET+CDT^44^. For bridge-text datasets, our baselines include DecomposedMeta-NER, ESD, ProtoShot, and StructShot.

### Evalution protocols

We followed the N-way K-shot downsampling setting proposed by Ding et al.^28^. This sampling strategy ensures that the sampling set contains N types of entities, and each type of entity appears $K \sim 2K$ times. We report the micro-F1 scores with standard deviations for the different baselines.

The calculation formula for the micro-F1 is as follows.

9 $$\begin{aligned} Micro-F1 = \frac{2 \times precision_{mi} \times recall_{mi}}{precision_{mi} + recall_{mi}} \end{aligned}$$

Where the calculation formula for $precision_{mi}$ and $recall_{mi}$ are as follows.

10 $$\begin{aligned} precision_{mi}= & \frac{\Sigma _{i=1}^n T P_i}{\Sigma _{i=1}^n T P_i + \Sigma _{i=1}^n F P_i} \end{aligned}$$

11 $$\begin{aligned} recall_{mi}= & \frac{\Sigma _{i=1}^n T P_i}{\Sigma _{i=1}^n T P_i + \Sigma _{i=1}^n F N_i} \end{aligned}$$

In formulas (10) and (11), $TP_{i}$ refers to True Positive for class *i* , which means that the positive instances in class *i* are correctly classified as positive. $FP_{i}$ refers to a False Positive for class *i*, which means that the negative instances in class *i* are incorrectly classified as positive instances. $FN_{i}$ refers to False Negative for class *i*, which means that the positive instances in class *i* are incorrectly classified as negative.

### Implemention details

We implemented our method using PyTorch version 1.13.1. We used AdamW^47^ for optimization and set the AdamW optimizer with a 10% linear warmup scheduler; the weight decay ratio was le-2. The value of the hyperparameter is chosen from 0.15, 0.35, 0.55, 0.75, 0.95 and is set to 0.75 by default (which is good enough for almost all cases). The learning rates of the encoder is 2e-4, and the learning rate of the decoder is 2e-3. For all datasets, we train UnionPromtNER for 50-100 epochs. We set the batch size to 1 to narrow the gap between training and fine-tuning, which means that we used one episode per step to update our model. We trained our model on the training set and used the validation set to select the model with the highest F1 scores.

## Results and analysis

### Main results

Tables 2, 4, and 5 report the performance of UnionPromptNER on the three different datasets. It can be observed that: 1) in the 20 sets of experiments, our method achieved the best performance in 19 sets. To compare with other SOTA methods, we collected the configurations of the other methods and calculated their average and maximum improvement values on the three different datasets. The results indicate that UnionPromptNER significantly outperforms the previous SOTA methods in terms of the micro-averaged F1 score, with an average improvement of 2.84% and a maximum improvement of 11.48%. These comparisons suggest that the proposed method is effective for few-shot NER. 2) in the two types of datasets in Few-NERD, our method shows a more significant performance improvement on Few-NERD Intra compared to Few-NERD Inter. From the results in Table 1, it can be seen that when facing challenging tasks, UnionPromptNER exhibits superior transfer learning capabilities compared with previous methods.

**Fig. 4:**
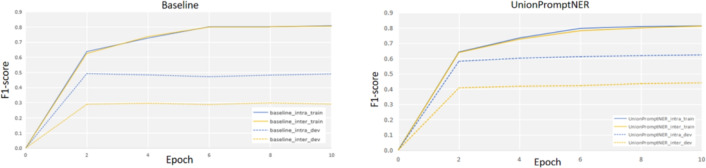
Training curves for CONTaiNER Das et al. (2022) baseline (on the left) and our model (on the right).The experiments are conducted under Few-NERD INTRA 1-shot and INTER 1-shot setting.

Our architecture of using multiple prompts also mitigates overfitting. We conduct two experiments on Few-NERD to prove this empirically. Figure 4 shows the training curves for CONTaiNER (Das et al., 2022) and our model. From the curves, it can be observed that the performance trends of both models on the training set are similar. However, the performance of CONTaiNER on the development set stops improving much earlier, while our model performs better in the later training epochs. Compared to CONTaiNER, our model shows significant improvement in the later stages. This indicates that in the few-shot setting UnionPromptNER is less affected by overfitting.

To compare whether there are significant differences in the metric data between the experimental group (UnionPromptNER) and the control groups (DecomposedMetaNER, ESD, CONTaiNER, StructShot, ProtoBERT, and NNShot), we conducted a paired t-test on the Intra data in Table 2, and the corresponding result data are presented in Table 3.

Among them, “U” in Table 3 refers to the UnionPromptNER proposed in this paper, while “D”, “E”, “C”, “S”, “P”, and “N” represent the DecomposedMetaNER, ESD, CONTaiNER, StructShot, ProtoBERT, and NNShot models respectively. It can be seen from the result data that compared with the aforementioned methods, the p-values of the UnionPromptNER method proposed in this paper are all far less than 0.05. That is, from the perspective of the result data, there are significant differences between the experimental group and the control groups, which indicates that the performance of UnionPromptNER is significantly better than that of the methods in the control groups.

**Table 2:** F1 scores with standard deviations on Few-NERD for both Inter and Intra settings.

Models	1 $\sim$ 2-shot		5 $\sim$ 10-shot		Avg.
	5 way	10 way	5 way	10 way	
Intra					
DecomposedMetaNER	48.32 ± 0.31	42.84 ± 0.46	62.92 ± 0.57	53.14 ± 0.25	52.10
ESD	36.08 ± 1.60	30.00 ± 0.70	52.17 ± 1.50	42.15 ± 2.60	40.09
CONTaiNER	40.43	33.84	53.70	47.49	43.87
StructShot	30.21 ± 0.90	21.03 ± 1.13	38.00 ± 1.29	26.42 ± 0.60	28.92
ProtoBERT	20.76 ± 0.84	15.05 ± 0.44	42.54 ± 0.94	35.40 ± 0.13	28.44
NNShot	25.78 ± 0.91	18.27 ± 0.41	36.18 ± 0.79	27.67 ± 1.06	26.98
UnionPromptNER	54.87 ± 0.27	49.28 ± 0.63	65.30 ± 1.01	59.63 ± 0.34	57.27
Inter					
DecomposedMetaNER	64.75 ± 0.35	58.65 ± 0.43	71.49 ± 0.47	68.11 ± 0.05	65.75
ESD	59.29 ± 1.25	52.16 ± 0.79	69.06 ± 0.80	64.00 ± 0.43	61.13
CONTaiNER	55.95	48.35	61.83	57.12	55.81
StructShot	51.88 ± 0.69	43.34 ± 0.10	57.32 ± 0.63	49.57 ± 3.08	50.53
ProtoBERT	38.83 ± 1.49	32.45 ± 0.79	58.79 ± 0.44	52.92 ± 0.37	45.75
NNShot	54.29 ± 0.40	46.98 ± 1.96	50.56 ± 3.33	50.00 ± 0.36	50.46
UnionPromptNER	64.83 ± 0.14	60.24 ± 0.64	71.65 ± 0.20	69.33 ± 0.31	66.51

**Table 3:** T-test Analysis Results.

Category	Experimental group	Control group	t	P
(U,D)	[54.87,49.28,65.3,59.63,57.27]	[48.32,42.84,62.92,53.14,52.1]	-6.7667	0.0025
(U,E)	[54.87,49.28,65.3,59.63,57.27]	[36.08,30.00,52.17,42.15,40.00]	-15.8576	0.0001
(U,C)	[54.87,49.28,65.3,59.63,57.27]	[40.43,33.84,53.7,47.49,43.87]	-18.8894	0
(U,S)	[54.87,49.28,65.3,59.63,57.27]	[30.21,21.03,38.00,26.42,28.92]	-20.4766	0
(U,P)	[54.87,49.28,65.3,59.63,57.27]	[20.76,15.05,42.54,35.4,28.44]	-12.0215	0.0003
(U,N)	[54.87,49.28,65.3,59.63,57.27]	[25.78,18.27,36.18,27.67,26.98]	-54.7815	0

**Table 4:** F1 scores with standard deviations on CrossNER.

Models	CoNLL03	GUM	WNUT	OntoNotes	Avg.
1-shot					
DecomposedMetaNER	45.83±0.25	16.34±0.33	25.01±0.68	34.76±0.24	30.49
SimBERT	19.22	6.91	5.18	13.99	11.32
TransferBERT	4.75±1.42	0.57±0.32	2.71±0.72	3.46±0.54	2.87
ProtoBERT	32.49±2.01	3.89±0.24	10.68±1.40	6.67±0.46	13.43
Matching Network	19.50±0.35	4.73±0.16	17.23±2.75	15.06±1.61	14.13
L-TapNET+CDT	44.30±3.15	12.04±0.65	20.80±1.06	15.17±1.25	23.08
UnionPromptNER	46.31±0.61	19.58±0.45	27.62±0.87	35.69±0.53	32.3
5-shot					
DecomposedMetaNER	58.04±0.63	30.68±0.46	31.03±2.04	45.61±0.77	41.34
SimBERT	32.01	10.63	8.20	21.14	18
TransferBERT	15.36±2.81	3.62±0.57	11.08±0.57	35.49±7.60	16.39
ProtoBERT	50.06±1.57	9.54±0.4 4	17.26±2.65	13.59±1.61	22.61
Matching Network	19.85±0.74	5.58±0.23	6.61±1.75	8.08±0.47	10.03
L-TapNET+CDT	45.35±2.67	11.65±2.34	23.30±2.80	20.95±2.81	25.32
UnionPromptNER	62.04±0.33	42.06±0.88	30.54±0.67	47.98±0.18	45.68

**Table 5:** F1 scores with standard deviations on Bridge Text Datasets.

Models	1 $\sim$ 2-shot		5 $\sim$ 10-shot		Avg.
	5 way	10 way	5 way	10 way	
DecomposedMetaNER	44.28	40.55	58.09	50.25	48.29
ESD	32.64	28.17	49.63	41.35	37.95
ProtoBERT	19.67	15.11	38.54	33.01	26.58
StructShot	28.94	20.96	36.51	24.55	27.74
UnionPromptNER	45.90	42.83	60.19	52.64	50.39

### Ablation study

In this section, we demonstrate the contributions of the different parts of UnionPromptNER. The ablation study results for the Few-NERD (including Intra and Inter), OntoNotes, and Bridge Text datasets are shown in Table 6.

**Table 6:** Ablation Study for UnionPromptNER. The variants M and N refer to using the fixed-template prompts and label-semantic prompts only, respectively. Plain refers to the original inputs and plain+N refers to replacing the fixed-template prompt with the original inputs. M+N is our UnionPromptNER method.

Setting	Models	Intra	Inter	OntoNotes	Bridge Text Datasets
5$\sim$10-shot	plain	48.32±0.31	54.32±0.54	42.17±0.79	55.27
	M($\rho$ = 1.0)	64.44±0.38	67.82±0.53	46.67±0.45	58.34
	N($\rho$ = 0.0)	58.10±1.29	56.31±1.67	43.48±1.05	55.57
	plain+N($\rho$ = 0.35)	59.04±0.55	58.46±0.17	42.68±1.32	55.97
	plain+N($\rho$ = 0.55)	60.04±0.64	61.34±0.87	42.77±0.42	56.14
	plain+N($\rho$ = 0.75)	60.38±0.41	61.88±0.77	43.66±0.07	56.84
	M+N($\rho$ = 0.35)	60.17±0.28	62.97±0.11	44.64±0.17	56.09
	M+N($\rho$ = 0.55)	61.06±0.46	64.61±0.98	44.07±0.41	57.61
	M+N($\rho$ = 0.75)	65.30±1.01	71.65±0.20	47.98±0.18	60.19

**Table 7:** The comparison results between three other prompt methods and the fixed-template prompts as well as the label-semantic prompts. The variants C refers to direct restore formula prompts, D refers to restore form with type explanation prompts, E refers to nested entity hierarchical restoration prompts.

Setting	Models	Intra	Inter	OntoNotes	Bridge text datasets
5$\sim$10-shot	plain	48.32±0.31	54.32±0.54	42.17±0.79	55.27
	M	64.44±0.38	67.82±0.53	46.67±0.45	58.34
	N	58.10±1.29	56.31±1.67	43.48±1.05	55.57
	C	51.45±0.89	54.89±0.17	42.08±0.22	54.38
	D	53.99±1.18	54.43±0.47	42.49±0.83	54.72
	E	52.59±0.74	53.89±0.62	41.55±0.38	53.61

**Fixed-Template Prompts & Label-Semantic Prompts** According to Table 6, based on the comparison between the prompt-based method and the non-prompt-based method, the former consistently outperforms the latter in terms of performance. These improvements align with the motivations discussed in previous sections. With the help of these two prompt methods, the model can better utilize the information provided and learn the representations for each token in the input statement.

**The Effectiveness of Masked Weighted Average** According to the results from Table 6, our UnionPromptNER demonstrates significantly better performance across all testing datasets compared to all baseline models, which indicates that both prompting methods contribute to the final performance of the model. By adjusting the value of the average weight $\rho$, the weights of the two representations for different data distributions can be balanced. Through multiple experiments, we found that the model performed optimally in most cases, when $\rho =0.75$. Therefore, 0.75 can be considered as the default hyperparameter in our framework. In addition, when other values of $\rho$, UnionPromptNER still demonstrated performance above most of the baseline models, indicating that our proposed framework is robust.

**Qualitative Error Analysis** We conducted a qualitative error analysis, taking the experimental results of the six baseline models in Table 2 and our proposed UnionPromptNER model on the Few-NERD dataset as examples. Due to the lack of relevant examples, models such as ProtoBERT, NNShot, and ESD struggled to understand the task requirements and were prone to semantic comprehension biases. When handling the task, the DecomposedMetaNER, CONTaiNER, and StructShot models failed to fully extract or utilize the key information in the input text, missing important attribute information of entities, which resulted in mediocre performance. In contrast, the UnionPromptNER proposed in this paper, by integrating the advantages of multiple prompt strategies, can consolidate the guidance of different prompt methods on key information, thereby reducing the occurrence of information omission errors and interference errors caused by redundant information.

**Case Study** We selected a small number of examples from the WNUT 1-shot dataset and Bridge Text dataset 1-shot to validate the prediction capabilities of the UnionPromptNER and CONTaiNER models. The results are presented in Tables 8 and 9. UnionPromptNER provides better predictions than CONTaiNER in most cases.

We also applied UnionNERPrompt to the dataset of the water transportation and water conservancy industry for testing, and the test results are shown in Table 10. It can be seen from Table 10 that compared with the CONTaiNER model, UnionNERPrompt also performs well on the dataset of the water transportation and water conservancy industry, with higher accuracy.

Similarly, we have tried other prompt construction methods: direct restore formula prompts (denoted as C), restore form with type explanation prompts (denoted as D), and nested entity hierarchical restoration prompts (denoted as E). The comparison results between these three methods and the fixed-template prompts as well as the label-semantic prompts are shown in Table 7. It can be seen from Table 7 that these three methods have the problems of insufficient accuracy and coverage.

**Table 8:** Case study: An illustration of some cases from the WNUT test set. In WNUT test set, there are 6 entities: person(PER), location(LOC), product(PRO), creative work(CW), miscellaneous(MIS), group(GRO). Here bold represents correct predictions, while italic represents mistakes.

Ground truth	UnionPromptNER	CONTaiNER
wow ${\textbf {emma}}_{{\textbf {PER}}}$ and ${\textbf {kaite}}_{{\textbf {PER}}}$ is so very cute and so funny i wish im ${\textbf {ryan}}_{{\textbf {PER}}}$	wow ${\textbf {emma}}_{{\textbf {PER}}}$ and ${\textbf {kaite}}_{{\textbf {PER}}}$ is so very cute and so funny i wish im ${\textbf {ryan}}_{{\textbf {PER}}}$	wow ${\textbf {emma}}_{{\textbf {PER}}}$ and ${\textbf {kaite}}_{{\textbf {PER}}}$ is so very cute and so funny i wish $im_{PER}$ ${\textbf {ryan}}_{{\textbf {PER}}}$
great video! good comparisons between the ${\textbf {ipad}}_{{\textbf {PRO}}}$ and the ${\textbf {ipad}}_{{\textbf {PRO}}}$ ${\textbf {pro}}_{{\textbf {PRO}}}$!	great video! good comparisons between $the_{PRO}$ ${\textbf {ipad}}_{{\textbf {PRO}}}$ and the ${\textbf {ipad}}_{{\textbf {PRO}}}$ ${\textbf {pro}}_{{\textbf {PRO}}}$!	great video! good comparisons between the *ipad* and the *ipad* ${\textbf {pro}}_{{\textbf {PRO}}}$!
i pronounce it nye-on cat	i pronounce it nye-on cat	i pronounce it $nye-on_{PER}$ $cat_{PRO}$

**Table 9:** Case study: An illustration of some cases from the Bridge Text test set. In Bridge Text test set, there are 5 entities: standard(STD), number(NUM), position(POS), disease(DIS), CMP(compare). Same as described above, bold indicates correct predictions, while italic indicates mistakes.

Ground truth	UnionPromptNER	CONTaiNER
The **“Standard for Engineering** ${\textbf {Surveying}}_{\textbf {STD}}$” has been approved as a national standard, with the code ${\textbf {GB50026-2020}}_{\textbf {NUM}}$, and will be implemented from June 1, 2021.	The **“Standard for Engineering** ${\textbf {Surveying}}_{\textbf {STD}}$” has been approved as a national standard, with the code ${\textbf {GB50026-2020}}_{\textbf {NUM}}$, and will be implemented from June 1, 2021.	The **“Standard for Engineering** ${\textbf {Surveying}}_{\textbf {STD}}$” has been approved as a national standard, with the code *GB50026-2020*, and will be implemented from June 1, 2021.
The main bridge’s **spherical tension-compression** ${\textbf {bearing}}_{\textbf {POS}}$ steel plate has **paint** ${\textbf {peeling}}_{\textbf {DIS}}$ and ${\textbf {corrosion}}_{\textbf {DIS}}$.	The main bridge’s **spherical tension-compression** ${\textbf {bearing}}_{\textbf {POS}}$ steel plate has *paint* *peeling* and ${\textbf {corrosion}}_{\textbf {DIS}}$.	The main bridge’s *spherical tension-compression bearing steel* plate has *paint peeling* and ${\textbf {corrosion}}_{\textbf {DIS}}$.
The **vertical line of the** ${\textbf {casing}}_{\textbf {POS}}$ should coincide with the **centerline of the** ${\textbf {pile}}_{\textbf {POS}}$, and the error **should not** ${\textbf {exceed}}_{\textbf {CMP}}$ 50mm.	The **vertical line of the** ${\textbf {casing}}_{\textbf {POS}}$ should coincide with the **centerline of the** ${\textbf {pile}}_{\textbf {POS}}$, and the error **should not** ${\textbf {exceed}}_{\textbf {CMP}}$ 50mm.	The *vertical line of the casing* should coincide with the *centerline of the pile*, and the error **should not** ${\textbf {exceed}}_{\textbf {CMP}}$ 50mm.

**Table 10:** Case study: An illustration of some cases from the Water Transportation and Water Conservancy Industry Text test set. In the test set, there are 4 entities: facility(FAC), terminology(TER), position(POS), institution(INS). Same as described above, bold indicates correct predictions, while italic indicates mistakes.

Ground truth	UnionPromptNER	CONTaiNER
The **water** ${\textbf {level}}_{\textbf {FAC}}$ of this ${\textbf {reservoir}}_{\textbf {TER}}$ needs to be lowered below the safe water level before the rainy season arrives.	The **water** ${\textbf {level}}_{\textbf {FAC}}$ of this ${\textbf {reservoir}}_{\textbf {TER}}$ needs to be lowered below the safe water level before the rainy season arrives.	The **water** ${\textbf {level}}_{\textbf {FAC}}$ of this $reservoir_{TER}$ needs to be lowered below the safe water level before the rainy season arrives.
The **waterway management** ${\textbf {department}}_{\textbf {INS}}$ is carrying out dredging operations on the ${\textbf {waterways}}_{\textbf {FAC}}$ in **the middle and lower reaches of the Yangtze** ${\textbf {River}}_{\textbf {LOC}}$.	The **waterway management** ${\textbf {department}}_{\textbf {INS}}$ is carrying out dredging operations on the $waterways_{FAC}$ in **the middle and lower reaches of the Yangtze** ${\textbf {River}}_{\textbf {LOC}}$.	The *waterway management* ${\textbf {department}}_{\textbf {INS}}$ is carrying out dredging operations on the $waterways_{FAC}$ in **the middle and lower reaches of the Yangtze** ${\textbf {River}}_{\textbf {LOC}}$.
The gate of the ${\textbf {sluice}}_{\textbf {FAC}}$ has a ${\textbf {leakage}}_{\textbf {TER}}$ problem and needs to be repaired in time.	The gate of the ${\textbf {sluice}}_{\textbf {FAC}}$ has a ${\textbf {leakage}}_{\textbf {TER}}$ problem and needs to be repaired in time.	The gate of the $sluice_{FAC}$ has a ${\textbf {leakage}}_{\textbf {TER}}$ problem and needs to be repaired in time.

### Limitations

Because of our proposed model’s reliance on prompts from large language models, the model is unable to retain span information, meaning that it cannot represent the span of words or phrases in the output of large language models. This requires the extraction of predicted entity types and spans from the output of the large language model through manually designed parsing strategies. However, when the sentence to be predicted contains many repeated words or phrases, the effectiveness of this solution is prone to local optima. This is a data contamination problem that current mainstream large language models temporarily cannot avoid when predicting specific natural language processing tasks, as the training data used by these models are not publicly disclosed.

Another limitation of this study is the interpretability of the model results. Despite the surprising new capabilities exhibited by large language models with increasing model size and training data, there is currently a limited understanding of the source and interpretability of these new capabilities. It remains unclear why these large language models perform well in downstream natural language processing tasks, even without explicit training. Another limitation of this work is that it does not provide guidance on how to obtain the optimal $\rho$ value that needs to be preset.

## Conclusion

In this paper, we introduce UnionPromptNER, a method for bridge domain few-shot named entity recognition via a union prompting strategy. Our approach uses a union prompt strategy to instruct Pre-trained language models to extract entities with specific classes. We tested UnionPromptNER under 20 settings and found that it substantially outperformed the previous SOTA results by an of 2.84% and a maximum of 11.48% in the relative gains of micro F1. Ablation studies were conducted to demonstrate the positive impact of multiple prompt schemas on the generalizability of our model. This study provides a novel, simple, and effective baseline for few-shot learning in NER.

This paper has some possible limitations and future work should address the following problems: 1)To enhance the proposed model, it is possible to incorporate a priori knowledge by utilizing emerging technologies such as sentence embedding and knowledge graph embedding. This is particularly advantageous as external knowledge bases for specific domains often include more comprehensive vocabulary definitions. 2)Although the proposed model can perform a few-shot NER bridge domain, joint entity and relation extraction should be further studied. In addition, because bridge documents contain many tables, efficient tabular information extraction is another important and challenging task that should be considered in the future.

In addition, based on NER-related research, intelligent knowledge base questions and answering solutions for bridge domain information extraction should be explored.

## Data Availability

The datasets used and/or analyzed during the current study are available from the corresponding author Shuangshuang Xu on reasonable request via e-mail jhyxss@163.com.

## Code availability

The code used for this study are published in https://github.com/SSX0629/bridge-pro-ml.
